# The Global Mental Health Assessment Tool-validation in Hindi: A validity and feasibility study

**DOI:** 10.4103/0019-5545.74305

**Published:** 2010

**Authors:** Vimal K. Sharma, Savita Jagawat, Aarti Midha, Anil Jain, Anil Tambi, Leena Kumari Mangwani, Bhawna Sharma, Parul Dubey, Vipin Satija, John R. M. Copeland, Peter Lepping, Steven Lane, Murali Krishna, Ashok Pangaria

**Affiliations:** 1 Cheshire and Wirral Partnership NHS Foundation Trust, Wirral, Cheshire, UK; 2 Department of Psychiatry, Santokba Durlabhji Memorial Hospital, Jaipur, India; 3 Department of Neurology and Psychiatry, SMS Medical College Hospital, Jaipur, India; 4 Liverpool University, UK; 5 Glyndŵ University and Betsi Cadwaladr University Health Board, Lancashire, UK; 6 Edge Hill University, Lancashire, UK

**Keywords:** GMHAT, mental health assessment, primary care mental health, psychiatric diagnosis

## Abstract

**Background:**

A computer-assisted interview, the Global Mental Health Assessment Tool-validation (GMHAT/PC) has been developed to assist general practitioners and other health professionals to make a quick, convenient, yet reasonably comprehensive standardized mental health assessment. GMHAT/PC has been translated into various languages including Hindi. This is the first study conducted in India, using the Hindi version GMHAT/PC of the series of studies assessing its validity in different cultures.

**Aim:**

The study aims to assess the feasibility of using a computer assisted diagnostic interview by health professionals and to examine the level of agreement between the Hindi version GMHAT/PC diagnosis and psychiatrists’ ICD-10 based clinical diagnosis.

**Design:**

Cross-sectional validation study.

**Setting:**

Psychiatric clinic of a General Hospital and an out patient (Neurology) clinic in the Teaching General Hospital in Jaipur, India.

**Materials and Methods:**

All consecutive patients attending the psychiatric out patient clinic were interviewed using GMHAT/PC and psychiatrists made a diagnosis applying ICD-10 criteria for a period of six weeks. A small sample of subjects was interviewed in a similar way in a Neurology clinic for four weeks.

**Results:**

The mean duration of interview was under 17 minutes. Most patients were pleased that they were asked about every aspect of their mental health. The agreement between psychologists’ GMHAT/PC interview diagnoses and psychiatrists’ clinical diagnoses was excellent (Kappa 0.96, sensitivity 1.00, and specificity 0.94).

**Conclusion:**

GMHAT/PC Hindi version detected mental disorders accurately and it was feasible to use GMHAT/PC in Indian settings.

## INTRODUCTION

Mental health problems are one of the leading causes of disability in the world (WHO 2001).[1] A vast majority of people with mental disorders including with severe mental illness view primary care as the cornerstone of their health care system.[2] It takes about six years to train a doctor and a further 6 years to train as a psychiatrist. The low and middle-income countries therefore have few doctors and even fewer psychiatrists, because of the high cost of medical education and the problems with retention of doctors once they have qualified. There is no foreseeable answer to this problem. As a result many thousands of mentally ill people remain untreated. The challenges of establishing satisfactory community mental health services in developing countries have been addressed by Murthy and Kumar highlighting the need for training on mental health to health workers and utilizing community resources in providing care to the mentally ill.[3]

Sharma and Copeland have developed a computer-assisted semi-structured, interviewer rated package, the Global Mental health Assessment Tool (GMHAT/PC), with primary care workers, which has already been translated into a number of languages of low- and middle-income countries. The package is an innovative way to address this problem. This method aims to improve the recognition of mental illness in primary care and initiation of appropriate treatments by skilling up primary care workers. The use of computers could be a restriction, but we are developing the program to be installed on a touch-screen PDA, making it easy to use anywhere. These methods which have so far taken 7 years to adapt and develop are based on many years of developing and using computer-assisted research diagnostic tools.

Our research so far demonstrated the feasibility of using this method in primary care and general health setting.[4–6] Patients on the whole received the GMHAT/PC assessment well and said they found it helpful as it covered more aspects of their mental health than the usual consultation. As it covers a wide range of mental disorders including psychoses and organic disorders should prove useful in their early and accurate detection.

The format of GMHAT/PC is simple to administer as questions appearing on the screen cover only one area. The interviewer would benefit from having some background experience of assessing mental health problems and requires little training to use the schedule. For those who have no previous experience of mental health assessments, a short training package would be necessary.

The description of the GMHAT/PC is outlined in the research reports,[4–7] which highlights its reliability and validity as well as its usefulness in primary care and general health setting. It also shows reliability and validity among different psychiatrists using HADS scores as a comparator. Its content is summarized here.

On computer, the first screen is for patient information and program administration followed by instruction page that gives details of how to use the tool and rate the symptoms. The following screens consist of questions on mental state symptoms or problems: worries; anxiety and panic attacks; concentration; depressed mood, including suicidal risk; sleep; appetite; eating disorders; hypochondriasis; obsessions and compulsions; phobia; mania/hypomania; psychotic symptoms; disorientation; memory impairment; alcohol misuse; drug misuse; personality problems; and stressors. The questions proceed in clinical order along a tree-branch structure. For each of the major clinical disorders there are key screening questions with cut off points thus economizing on the time. The interviewer may provide a clinical diagnosis in the next section. A summary report of symptoms, their scores and a GMHAT/PC diagnosis is produced in a printable form. The main computer diagnosis is derived using a hierarchical model and designed around ICD-10.[8] The diagnostic program takes account of the severity of symptoms (moderate to severe). It also generates alternative or additional diagnoses based on the presence of symptoms of other disorders. In addition, it includes an assessment of risk of self-harm. The program also contains management guidelines for these disorders.

The Hindi version was developed using the standard methodology applied in all other versions of GMHAT/PC. The English version was translated into Hindi, was back translated by an independent translator, and compared and judged by a panel of experts. Further minor modifications were made following field trials with full approval of the panel of experts.

## MATERIALS AND METHODS

The study was planned in two settings in Jaipur, India, in order to include persons with varying degree of mental health problems. First one was Psychiatric Out-Patient Clinic of a large general hospital, Santokba Durlabhji Memorial Hospital (SDMH), the other one was the Neurology Out-Patient Clinic of SMS Hospital (SMSH). All consecutive patients in the Psychiatric Out-patient Clinic for a period of 6 weeks and in the Neurology Clinic for a period of 4 weeks were included in the study. The study was approved by their respective ethical committees. All patients were informed about the purpose of the study. Only those who gave valid written informed consent participated in the study. The interviewers had a brief training session to familiarize themselves with the GMHAT/PC and how to rate symptoms before interviewing patients. Two clinical psychologists (SJ and LKM) interviewed patients at SDMH and SMSH clinics. The psychiatrists (AM and AT), unaware of the GMHAT ratings and diagnosis, interviewed the patients immediately afterwards and recorded her/his independent clinical diagnosis based on ICD-10 clinical criteria. Demographic data, computer-generated (GMHAT/PC) diagnoses, and the psychiatrist’s clinical diagnoses were recorded on the database.

### Statistics

The kappa coefficient was used to determine the levels of agreement between the GMHAT/PC-assisted psychologists’ interview diagnoses and the psychiatrist’s diagnoses (gold standard). Sensitivity and specificity analysis was then used to determine how the GMHAT/PC-assisted interviews could identify the cases with and without mental illness as determined by the psychiatrists.

## RESULTS

Eighty-two patients participated in the study, 49 (60%) males and 33 (40%) females in the age range of 13--68 with a mean age of 36.5 years. There was no significance difference between the gender groups with regards to age or time taken to complete GMHAT/PC. The overall mean time taken to administer GMHAT/PC) was 16.3 min (median 16.5, range 5-35 min).

None of the patients declined their consent to participate in the study.

### Validity of GMHAT

There is good level of agreement between the psychologists’ (GMHAT/PC) diagnoses and the psychiatrists’ (clinical) diagnoses of any mental illness, Kappa 0.96, 95% C.I. (0.89, 1.00). There is good sensitivity (0.94) and specificity (1.0), with psychologists correctly identifying 64 out of the 65 participants diagnosed with mental illness and 16 out of 16 of those without it.

The level of agreement for the diagnoses of neurotic illnesses was good; Kappa 0.90, 95% C.I. (0.78, 1.00). Sensitivity was 0.85 with psychologist correctly identifying 17 of the 20 participants diagnosed with neurosis. The specificity was 1.0, with the psychologists correctly identifying 62 of the 62 participants not suffering from neurosis.

The level of agreement for depression shows Kappa 0.85, 95% C.I. (0.73, 0.97). Sensitivity (0.91) and specificity (0.94) with the psychologists correctly identifying 31 of the 34 participants diagnosed by the psychiatrists as suffering from depression and 45 out of 48 of those without it.

The cases of psychosis were limited to establish sensitivity (0.83) and specificity (0.97) in a robust way; however 5 of the 6 cases were correctly identified by the psychologists using GMHAT/PC and 74 of 76 without it.

### Cases with disagreement

Overall only 7 out of 82 cases showed differences in GMHAT/PC and the psychiatrists’ clinical diagnoses [Table 1]. Of the three cases of depression diagnosed by the psychiatrist, one had no primary GMHAT/PC diagnosis but had other possible diagnosis of depression. The remaining two had one organic and the other one psychosis as their GMHAT/PC primary diagnosis, but both had depression as additional possible diagnosis. The only one out of six psychosis cases diagnosed by psychiatrists was diagnosed by GMHAT/PC as a case of depression. This was a case that recovered from psychosis but had residual depressive symptoms. Of the 3 out of the 20 cases diagnosed by the psychiatrists as cases of neurosis where there were differences in their GMHAT/PC diagnosis, 2 were identified as cases of depression and 1 of psychosis by GMHAT/PC.

**Table 1:** Psychiatrists’ and GMHAT/PC (Psychologists’) diagnosis cross tabulation GMHAT/PC diagnosis

Psychiatrist’s clinical diagnosis	No.	Organic	Psychosis	Depression	Neurosis/stress	Other	Total
No	16						16
Organic		1					1
Psychosis			5	1			6
Depression	1	1	1	31			34
Neurosis/stress			1	2	17		20
Other						5	5
Total	17	2	7	34	17	5	82

### Feasibility

None of the subjects declined to participate in the study and all gave positive feedback. Most of them expressed satisfaction that the psychologists covered all aspects of their mental health using GMHAT/PC. Most patients and specially their relatives felt that they were under “quality and refined” assessment. Many patients requested to get the print out of the interview report. One example of the feedback is where the family member of the patient felt that by using GMHAT/PC interview his relative got the correct diagnosis and right treatment. The patients and relatives, who were seen in the clinics after the study was completed, came to know about the GMHAT/PC study and requested whether they could also have a “computer test” for diagnosis. The psychologists who interviewed patients found GMHAT/PC a very useful training tool to detect mental disorders.

## DISCUSSION

The findings of this study are encouraging and appear to support the view that other health professionals such as psychologists, and possibly others with some training can use the computer-assisted program GMHAT/PC in different cultures in making a valid assessment and diagnosis of mental disorders. Our previous study carried out in the UK proved that nurses can use GMHAT/PC in detecting mental disorders accurately in primary care and general health setting.[4,5] It was reassuring to find that the GMHAT/PC questions in Hindi were easy to understand by all the subjects interviewed from rural as well as urban area to detect psychopathology in that population. The mean duration of the interview of around 16 min, similar to our earlier UK studies,[4–6] makes it feasible in routine assessments in primary care and general health settings in India. The GMHAT/PC interviews were very well received by the subjects as well the interviewers. It was a novelty in the clinic and some other patients requested that their mental health should also be checked by the computer assessment.

Our findings are encouraging that the GMHAT/PC can help health professionals in detecting mental disorders in low- and middle-income countries. High mental health morbidity has a particular adverse effect on general health and social wellbeing in the population of developing countries.[9] Given that the identification of mental health problems is vital to improve the outcome in many chronic illnesses it is essential to improve their recognition rates. In their extensive review Patel *et al*. highlighted that common mental health conditions such as depression, schizophrenia, alcohol misuse, etc, can be treated effectively in low- and middle-income countries.[10] However, the lack of adequate mental health resources to deal with such a vast problem in India remains a challenge which may partially improve with the help of the voluntary sector.[11] A tool such as GMHAT/PC can assist health and voluntary sector workers in detecting and managing mental health conditions, using its pathways of care, derived from evidence-based guidelines such as NICE. It also adds to the skills of primary care health workers in detecting mental disorders more accurately. Murthy and Wig[12] highlighted the complexities of assessing mental health problems in countries such as India in their review of psychiatric diagnosis and classification in developing countries. Standardized assessments and diagnostic tools for common mental disorders in local languages for routine clinical use are hardly available, though authors acknowledge that some ICD-10 based specific disorder schedules such as for personality disorders IPDE[13] have been developed in Hindi. In addition to assist in diagnosing mental disorders, the GMHAT/PC would also help in planning evidence-based treatments, as the pathways of care and guidelines are part of the program. This will give more chance to follow the treatment guidelines compared to current practice.[14] The use of computers in routine clinical practice is still a challenge in low to middle income countries. However, diminishing costs has led to increased availability and use of computers even in remote areas of India. Our experience of using GMHAT/PC in this study suggests that its use in routine practice can be cost-effective for the following reasons. It takes on an average about 15 minutes to cover all common mental disorders, and in that it records necessary information that leads to useful clinical output. Any mental health care professional (not necessarily psychiatrists) with adequate training can use it. Therefore, GMHAT/PC could be a useful tool in implementing mental health programs in India.

### Strengths and limitations of the study

A sample with varying degrees of psychopathology in different health care settings is the strength of this study. The health care system in India is significantly different from Europe as there are no integrated primary health services and a significant number of people rely on private health care providers. It was therefore pragmatic to use the settings where psychologists and psychiatrists were available to carry out the study. We would have liked a higher number of cases included in the study, but the demand on services in both settings was such that we had to keep a realistic target.
